# Associations between oral behaviors, temporomandibular disorder subtypes and psychological distress in adult women: a retrospective case-control study

**DOI:** 10.22514/jofph.2024.030

**Published:** 2024-09-12

**Authors:** Yu Zhong, Fang Luo, Xinbei Li, Shiya Zeng, Shuyuan Zhang, Jiarui Si, Xin Xiong, Shanbao Fang

**Affiliations:** ^1^Guangxi Key Laboratory of Oral and Maxillofacial Rehabilitation and Reconstruction, Guangxi Stomatological Virtual Reality Engineering Research Center, College & Hospital of Stomatology, Guangxi Medical University, 530021 Nanning, Guangxi, China; ^2^State Key Laboratory of Oral Diseases & National Center for Stomatology & National Clinical Research Center for Oral Diseases, West China Hospital of Stomatology, Sichuan University, 610041 Chengdu, Sichuan, China; ^3^West China Hospital, Sichuan University, 610041 Chengdu, Sichuan, China

**Keywords:** Oral behaviors, Psychological distress, Temporomandibular joint disorders, Bruxism, TMD subgroups

## Abstract

Oral behaviors and psychological distress are known to be related to temporomandibular disorders (TMDs). However, the relationship between various oral behaviors and specific TMD subgroups in adult women experiencing psychological distress is still unclear. To investigate the relationship between various oral behaviors and different TMD subgroups with different psychological distress states. A total of 210 female TMD patients were divided into 3 subgroups according to their symptoms: pain-related (PT), intra-articular (IT) and combined pain-related and intra-articular (CT). Another 70 participants without TMDs were recruited as the non-TMD (NT) control group. We used reduced Chinese versions of the Oral Behavior Checklist (OBC-Ch 8), including awake (OBC-Ch 6) and sleep-related activities, the 9-item Patient Health Questionnaire (PHQ-9) and the 7-item Generalized Anxiety Disorder Scale (GAD-7) to assess oral behaviors and psychological distress. Differences in OBC scores among TMD subgroups were analyzed using Chi-square, Kruskal-Wallis H and *post hoc* tests, with significance set at *p* < 0.05. Oral behavior subscale scores significantly differed among TMD subgroups (*p *< 0.01). The OBC-Ch 8 scores of PT, IT and CT subjects were significantly higher than the NT group. PT and CT groups also had significantly higher GAD-7 and PHQ-9 scores. Sleep-related OBC scores of the PT, IT and CT groups were higher than those in the NT group, regardless of psychological states (*p *< 0.001). In psychologically distressed subjects, OBC-Ch 8 scores for PT and CT subgroups were significantly higher than those in the NT group. Oral behaviors are differentially associated with various TMD subgroups in female adults, and a correlation exists between individual psychological status and OBC scores.

## 1. Introduction

Temporomandibular disorders (TMDs) are a group of heterogeneous musculoskeletal conditions that involve the temporomandibular joints (TMJs), masticatory muscles and adjacent tissues, with a high prevalence among the general population in the modern world [1, 2]. Multiple etiological factors such as anatomy, trauma, genetic and psychosocial stress have been identified to contribute to the development of TMDs [3, 4].

As a component of the Diagnostic Criteria for TMD (DC/TMD) validation study, the Oral Behavior Checklist (OBC) was developed as a self-administered questionnaire to assess self-reported oral behaviors that are correlated with TMDs [5]. The OBC is the most widely accepted self-reporting tool that can be used to comprehensively assess various types of oral overuse behavior. Further, the OBC also is a good tool for evaluation of memory regarding unconscious oral behaviors [6, 7]. However, according to previous studies, OBC scores have not shown conclusive results related to TMDs. Some researchers have concluded that there is a correlation between painful TMDs and OBC scores [8, 9]. However, other studies have found no significant correlation between TMDs and OBC scores [10, 11]. To better assess patients’ oral behaviors, dentists and researchers usually combine self-reported OBC questionnaires with clinical examination including extraoral and intraoral evaluation [12].

Few studies have investigated the relationship between oral behaviors and specific TMD subgroups. Several studies have shown that some oral behaviors like tooth grinding, clenching, gum chewing and object biting are predictive of TMD risk [13]. Donnarumma *et al*. [14] analyzed OBC responses with exploratory factor analysis and classified OBC items into two groups. The first group included items pertaining to non-functional activities (NFA), such as clenching, grinding and holding. The other group included items pertaining to normal functional activities (FA) of the jaws, such as chewing, talking and yawning. The study explored the relationship between awake-state NFA and various TMD subgroups, and found that NFA scores in the painful TMD subgroup and the painful-dysfunctional TMD subgroup were higher than in the TMD-free control group.

There is general consensus that a biopsychosocial model should be applied to TMDs, and that patients with TMDs should be assessed in a manner that takes into account both physical and psychosocial factors [15]. A significant body of research suggests that psychological factors are strongly implicated in TMDs [16, 17, 18]. Several psychological variables can predict increased risk of TMD onset and persistence, including psychosocial stress, somatic symptoms and affective distress. Moreover, psychological characteristics such as anxiety and depression have been found to be related to different symptoms of TMDs [19, 20]. However, Donnarumma *et al*. [21] found that trait anxiety was only weakly related to the frequency of oral behaviors in healthy female individuals.

Bruxism is an umbrella term including various motor activities that may occur while conscious or unconscious. An expert consensus paper further clarified this distinction, and proposed separate definitions for awake bruxism (AB) and sleep bruxism (SB) [22]. The relationship between bruxism and TMD is still controversial and remains unclear. The majority of studies in which bruxism has been correlated with TMDs were conducted by means of a questionnaire or self-reported assessment. These papers have suggested a statistically significant association between bruxism and TMD [23]. Nevertheless, several other studies have identified bruxism based on clinical findings such as tooth wear or electromyographical measures, and the results did not support an association between bruxism and TMDs [24, 25]. Thus, the difference between effects of AB and SB on TMDs is still not clear.

Therefore, it is necessary to explore the relationship between oral behaviors in adult women with various TMD subtypes who are experiencing different psychological distress states. The aims of this study are as follows: (1) To assess the mean score oral behaviors in no TMDs and TMDs subgroups. (2) To study the association between TMD symptoms and bruxism. (3) The distribution of various oral behaviors with or without anxiety and depression in the no TMDs and TMDs subgroups.

In the present study, we take the null hypothesis to be that there is no significant difference in oral behavior scores among TMD subgroups, and that measures of oral behaviors are not correlated with TMD subgroups in women experiencing different types of psychological distress.

## 2. Materials and methods

### 2.1 Subjects

The study group consisting of female participants with TMDs was recruited from the Department of Temporomandibular Joint, West China Hospital of Stomatology. Enrollment lasted from 01 February 2022 to 30 March 2023. The healthy, non-TMD volunteers were recruited from the local community through public postings. The study only included female participants, in consideration of the high prevalence of TMDs among females [26]. The inclusion criteria were as follows: (1) age ≥18 years old; (2) capable of reading, comprehending and completing the questionnaires. Subjects were excluded from the study if they reported a history of TMD treatment; systemic diseases such as rheumatoid arthritis, tumor or trauma affecting the TMJs; or pharmacotherapy influencing TMD symptoms, including nonsteroidal anti-inflammatory drugs, anxiolytics and antidepressants.

### 2.2 Questionnaires

All the participants completed questionnaires to assess baseline variables, including a screen for five TMD symptoms (5Ts), abbreviated eight-item version of the Chinese translation of Oral Behaviors Checklist (OBC-Ch 8), 9-item Patient Health Questionnaire (PHQ-9) and 7-item Generalized Anxiety Disorder Scale (GAD-7), all of scales were translated into Chinese.

### 2.3 TMD subtypes and symptoms

All participants were asked to complete the 5Ts screening questionnaire to distinguish their subtype of TMD. The 5Ts showed excellent validity, sensitivity, reliability and specificity for identifying pain-related and/or intra-articular TMDs [27]. In the current study, clinical examination for Axis I DC/TMD diagnosis was administered by specialists in the Department of TMJ. Based on their questionnaire responses and physical examination, the subjects were categorized into 4 groups:

(1) Pain-Related TMD (PT) Group: pain-related TMD subtype, which included myalgia, arthralgia or headache attributed to TMDs.

(2) Intra-Articular TMD (IT) Group: intra-articular TMD symptoms, which included disc displacement with reduction and intermittent locking; disc displacement without reduction, with or without limited opening; degenerative joint disease or subluxation.

(3) Combined TMD (CT) Group: TMDs with both pain-related and intra-articular characteristics.

(4) Non-TMD (NT) group: Healthy subjects in the control group with no history of TMD diagnosis, matched in age with the other groups.

### 2.4 Oral behaviors and psychological distress

The participants completed OBC-Ch 8 scale. Eight items including six awake behaviors and two sleep behaviors were chosen to identify and quantify the overuse of the joints and muscles, according to the previous studies [8, 28]. The reliability of the Chinese version of OBC was shown to be good [7]. They answered six items (OBC-Ch 6) about awake oral behaviors, of which four concerned AB specifically, scored on a 5-point scale (1 = “none of the time” to 5 = “all of the time”). The questionnaire also included two items (OBC 1, 2) about asleep oral behaviors, one of them about SB, scored on a five-point scale (1 = “none of the time”, 2 = “less than 1 night per month”, 3 = “1 to 3 nights per month”, 4 = “1 to 3 nights per week”, 5 = “4 to 7 nights per week”). Only in patients reporting frequent bruxism was this behavior considered to be present. Thus, in this study, an OBC value ≥4 on any of the five questions about AB or SB was chosen as the conservative cut-off value for prevalence of AB or SB according to the previous study [29]. Total scores of awake (OBC-Ch 6) and all oral behaviors (OBC-Ch 8) were calculated by summing the OBC items. These items were selected for the study because these oral functional activities might be differentially associated with painful and dysfunctional TMD subtypes [14].

Psychological characteristics of all the subjects were assessed using Chinese versions of the validated PHQ-9 and GAD-7 self-reporting questionnaires. PHQ-9 was used to screen for depression, and items were rated based on the frequency of each symptom during the past two weeks, ranging from 0 (not at all) to 3 (nearly every day), with possible scores ranging from 0 to 27. In this study, the cut-off value for diagnosis of depression was ≥5. GAD-7, which assessed the severity of anxiety, was rated on a Likert scale from 0 (not at all) to 3 (nearly every day), resulting in a range of scores from 0 to 21. The cut-off value for diagnosis of anxiety was set to ≥5 in the present study [20].

### 2.5 Statistical analysis

The PASS Statistics package version 15.0 (NCSS, Kaysville, UT, USA) was used for sample size calculation. The power analysis determined that a minimum of 180 individuals should be included, with at least 45 participants in each group, for a medium effect size of Cohen’s *f* = 0.25, α = 0.05 and 1 − β = 0.80.

Continuous variables were described using the mean, standard deviation (SD), median and interquartile range (IQR). Categorical variables were expressed as the number of cases and percentage values. Kolmogorov-Smirnov tests were performed to check the normality of the data. Kruskal-Wallis H test was used for continuous variables with non-normal distribution and grade data. A Chi-square test or Fisher exact probability method was used to investigate categorical variables. Statistical analysis was performed using IBM SPSS v. 26.0 (IBM Corp., Armonk, NY, USA) and a level of *p* < 0.05 was established to determine statistical significance.

## 3. Results

The characteristics of the participants involved in the study are presented in Table 1. Each group consisted of 70 subjects, for a total of 280 participants with a mean age of 31.29 ± 10.36 included in the study. No significant difference was found in demographic variables (age and level of education) among the four groups (Table 1).

**Table 1.** Demographic characteristics of the different TMD groups.

Parameter		NT (n = 70)	PT (n = 70)	IT (n = 70)	CT (n = 70)	Total (n = 280)	*p*-value *Post-hoc*
Demography							
Age (yr)							
	Min–Max	18–55	18–68	18–66	18–62	18–68	0.309^b^
	Mean (SD)	31.9 (9.0)	32.1 (11.0)	30.1 (9.5)	31.1 (10.4)	31.3 (10.4)	
Education (%)							
	Primary or high school	19 (31.7%)	11 (18.3%)	12 (20.0%)	18 (30.0%)	60 (100.0%)	0.136^a^
	Undergraduate	48 (24.1%)	52 (26.1%)	49 (24.6%)	50 (25.1%)	199 (100.0%)	
	Graduate student	3 (14.3%)	7 (33.3%)	9 (42.9%)	2 (9.5%)	21 (100.0%)	
Psychological assessment							
GAD-7 score							
	Mean (SD)	2.4 (4.0)	5.2 (5.0)	3.4 (3.7)	5.5 (4.8)	4.1 (4.6)	<0.001^b^* PT, CT > NT; IT > CT
	Median (IQR)	0.0 (4.0)	4.0 (5.0)	2.0 (4.0)	4.0 (7.0)	3.0 (6.0)	
Anxiety (%)		16 (22.9%)	31 (44.3%)	20 (28.6%)	32 (45.7%)	99 (35.4%)	0.008^a^* PT, CT > NT
PHQ-9 score							
	Mean (SD)	2.7 (3.6)	5.5 (5.5)	3.7 (3.8)	5.2 (4.8)	4.3 (4.6)	<0.001^b^* PT, CT > NT
	Median (IQR)	1.0 (4.0)	4.0 (7.0)	3.0 (5.0)	4.0 (8.0)	3.0 (6.0)	
Depression (%)		16 (22.9%)	33 (47.1%)	22 (31.4%)	34 (48.6%)	105 (37.5%)	0.00^a^3* PT, CT > NT

^a^Chi-square test; ^b^Kruskal-Wallis-test; *Significantly different (GAD-7 score, Anxiety, PHQ-9 score, Depression) among the groups. Abbreviations: NT, no TMD; PT, pain-related TMD; IT, intra-articular TMD; CT, combined TMD; SD, standard deviation; IQR, interquartile range; GAD-7, the 7-item Generalized Anxiety Disorder Scale; PHQ-9, the 9-item Patient Health Questionnaire. Adjusted p value using Bonferroni correction.

Fig. 1 presents the AB and SB activity of each group. The prevalence of AB in the three TMD subtype groups was higher than in the NT group, though without significant difference (Fig. 1A). The frequency of AB ranged from 20% to 38.6% for the groups in our study. The prevalence of SB was significantly different among the groups (*p* < 0.001, Chi-square text), with the highest prevalence in the CT group (30%), followed by the PT group, IT group and NT group (Fig. 1B).

**Fig. 1.** The distribution of frequency of bruxism (n = 280). (A) The prevalence of AB and (B) SB in NT, PT, IT and CT groups. NT, no TMD; PT, pain-related TMD; IT, intra-articular TMD; CT, combined TMD; AB, awake bruxism; SB, sleep bruxism. *The prevalence of SB was significantly different among the groups.

There was a significant difference in oral behavior scores between the TMD subgroups and the NT group (*p* < 0.001). The total OBC-Ch 8 scores of the TMD subgroups were higher than that of the NT group (2.2 ± 3.0), as shown in Table 2. *Post hoc* pair-wise comparisons revealed that the OBC-Ch 8 scores of the PT group (11.5 ± 4.7), IT group (9.2 ± 5.6) and CT group (10.8 ± 5.2) were significantly higher than that of the NT group (2.6 ± 3.3) (Table 2).

**Table 2.** OBC scores in various TMD groups.

Parameter		NT (n = 70)	PT (n = 70)	IT (n = 70)	CT (n = 70)	*p*-value *Post-hoc*
OBC 1. Clench or grind teeth when asleep, based on any information you may have						
	Mean (SD)	1.1 (0.3)	2.4 (1.4)	2.3 (1.4)	2.4 (1.6)	<0.001^b^* PT, IT, CT > NT
	Median (IQR)	1.0 (0.0)	2.0 (2.0)	2.0 (2.0)	2.0 (3.0)	
OBC 2. Sleep in a position that puts pressure on the jaw (for example, on stomach, on the side)						
	Mean (SD)	1.2 (0.5)	4.1 (1.1)	3.9 (1.6)	3.9 (1.4)	<0.001^b^* PT, IT, CT > NT
	Median (IQR)	1.0 (0.0)	4.0 (2.0)	5.0 (2.0)	4.5 (2.0)	
Sleep-related OBC total						
	Mean (SD)	0.3 (0.7)	4.5 (1.9)	4.2 (2.5)	4.3 (2.3)	<0.001^b^* PT, IT, CT > NT
	Median (IQR)	0.0 (0.0)	4.5 (3.0)	4.0 (3.3)	4.0 (3.0)	
OBC 3. Grind teeth together during waking hours						
	Mean (SD)	1.3 (0.8)	1.4 (0.7)	1.2 (0.8)	1.2 (0.6)	0.500
	Median (IQR)	1.0 (0.0)	1.0 (0.0)	1.0 (0.0)	1.0 (0.0)	
OBC 4. Clench teeth together during waking hours						
	Mean (SD)	2.0 (1.5)	2.5 (1.1)	1.9 (1.0)	2.3 (1.3)	<0.001^b^* PT > NT, IT
	Median (IQR)	1.0 (1.0)	2.5 (1.0)	2.0 (2.0)	2.0 (2.0)	
OBC 5. Press, touch or hold teeth together other than while eating (that is, contact between upper and lower teeth)						
	Mean (SD)	1.1 (0.3)	2.8 (1.1)	2.5 (1.4)	2.7 (1.2)	<0.001^b^* PT, IT, CT > NT
	Median (IQR)	1.0 (0.0)	3.0 (2.0)	2.0 (3.0)	3.0 (1.0)	
OBC 6. Hold, tighten or tense muscles without clenching or bringing teeth together						
	Mean ± SD	1.2 (0.5)	2.2 (1.0)	1.7 (0.9)	2.0 (1.0)	<0.001^b^* PT, IT, CT > NT; PT > IT
	Median (IQR)	1.0 (0.0)	2.0 (2.0)	1.5 (1.0)	2.0 (2.0)	
OBC 7. Hold or jut jaw forward or to the side						
	Mean (SD)	1.4 (0.9)	1.9 (0.9)	1.6 (0.8)	2.1 (1.1)	<0.001^b^* PT, CT > NT; PT > IT
	Median (IQR)	1.0 (0.0)	2.0 (1.0)	1.0 (1.0)	2.0 (2.0)	
OBC 11. Hold jaw in rigid or tense position, such as to brace or protect the jaw						
	Mean ± SD	1.2 (0.5)	2.2 (1.0)	2.0 (1.1)	2.2 (0.9)	<0.001^b^* PT, IT, CT > NT
	Median (IQR)	1.0 (0.0)	2.0 (2.0)	2.0 (2.0)	2.0 (2.0)	
OBC-Ch 6						
	Mean (SD)	2.2 (3.0)	7.0 (3.9)	5.0 (3.9)	6.6 (4.0)	<0.001^b^* PT, IT, CT > NT; PT > IT
	Median (IQR)	1.0 (4.0)	7.0 (6.0)	5.0 (6.0)	6.0 (5.0)	
OBC-Ch 8						
	Mean (SD)	2.6 (3.3)	11.5 (4.7)	9.2 (5.6)	10.8 (5.2)	<0.001^b^* PT, IT, CT > NT
	Median (IQR)	1.0 (4.0)	12.0 (6.0)	9 .0(8.0)	10.0 (6.0)	

^b^Kruskal-Wallis-test; *There was a significant difference in oral behavior scores between the TMD subgroups and the NT group. Abbreviations: NT, no TMD; PT, pain-related TMD; IT, intra-articular TMD; CT, combined TMD; OBC, Oral Behavior Checklist; OBC-Ch, Chinese versions of the Oral Behavior Checklist; SD, standard deviation; IQR, interquartile range. Adjusted p value using Bonferroni correction.

To compare the psychological factors of DC/TMD Axis II, the average scores on the GAD-7 and PHQ-9 were calculated. Table 1 demonstrated that the average GAD-7 and PHQ-9 scores were above the preselected cut-off value only in the PT group (5.2 ± 5.0, 5.5 ± 5.5) and CT group (5.5 ± 4.8, 5.2 ± 4.8). The prevalence of anxiety and depression based on our diagnostic threshold was significantly higher in the TMD subgroups collectively than in the NT group (*p* < 0.01). *Post hoc* pair-wise comparisons indicated that the scores of the PT and CT groups were each significantly higher than that of the NT group (Table 1).

Further, comparison of the psychological characteristics of each TMD subgroup in Tables 3 and 4 revealed that the scores of sleep-related OBC in the three TMD subgroups were each higher than that of the NT group (*p* < 0.001).

**Table 3.** Oral Behavior Checklist (OBC) scores disaggregated by anxiety status.

Parameter		Anxiety (n = 99)					No anxiety (n = 181)				
		NT (n = 16)	PT (n = 31)	IT (n = 20)	CT (n = 32)	*p*-value *Post-hoc*	NT (n = 54)	PT (n = 39)	IT (n = 50)	CT (n = 38)	*p*-value *Post-hoc*
OBC 1. Clench or grind teeth when asleep, based on any information you may have											
	Mean (SD)	1.3 (0.4)	2.3 (1.4)	2.4 (1.2)	2.7 (1.6)	0.007* IT, CT > NT	1.1 (0.3)	2.5 (1.3)	2.3 (1.5)	2.2 (1.6)	<0.001^b^* PT, IT, CT > NT
	Median (IQR)	1.0 (1.0)	2.0 (2.0)	2.0 (2.0)	3.0 (3.0)		1.0 (0.0)	2.0 (3.0)	2.0 (2.0)	1.0 (3.0)	
OBC 2. Sleep in a position that puts pressure on the jaw (for example, on stomach, on the side)											
	Mean (SD)	1.5 (0.7)	4.0 (1.2)	3.6 (1.7)	4.3 (1.1)	<0.001^b^* PT, IT, CT > NT	1.2 (0.4)	4.1 (1.1)	4.0 (1.5)	3.6 (1.6)	<0.001^b^* PT, IT, CT > NT
	Median (IQR)	1.0 (1.0)	5.0 (2.0)	5.0 (3.0)	5.0 (1.0)		1.0 (0.0)	4.0 (1.0)	5.0 (2.0)	4.0 (3.0)	
Sleep-related OBC total											
	Mean (SD)	0.8 (1.0)	4.4 (2.2)	4.0 (2.5)	5.0 (2.2)	<0.001^b^* PT, IT, CT > NT	0.2 (0.6)	4.6 (1.7)	4.3 (2.5)	3.7 (2.3)	<0.001^b^* PT, IT, CT > NT
	Median (IQR)	0.0 (1.8)	4.0 (4.0)	4.0 (4.8)	5.5 (3.5)		0.0 (0.0)	5.0 (3.0)	4.0 (3.0)	4.0 (3.3)	
OBC 3. Grind teeth together during waking hours											
	Mean (SD)	1.7 (0.9)	1.4 (0.8)	1.2 (0.4)	1.2 (0.5)	0.043	1.2 (0.7)	1.3 (0.6)	1.3 (0.9)	1.3 (0.6)	0.288
	Median (IQR)	1.5 (1.0)	1.0 (0.0)	1.0 (0.0)	1.0 (0.0)		1.0 (0.0)	1.0 (1.0)	1.0 (0.0)	1.0 (0.0)	
OBC 4. Clench teeth together during waking hours											
	Mean (SD)	3.2 (1.6)	2.7 (1.2)	2.2 (1.0)	2.6 (1.3)	0.204	1.6 (1.2)	2.4 (1.1)	1.8 (0.9)	2.1 (1.2)	<0.001^b^* PT, CT > NT
	Median (IQR)	3.0 (3.0)	3.0 (1.0)	2.0 (2.0)	2.5 (1.0)		1.0 (1.0)	2.0 (1.0)	2.0 (1.0)	2.0 (2.0)	
OBC 5. Press, touch, or hold teeth together other than while eating (that is, contact between upper and lower teeth)											
	Mean (SD)	1.2 (0.4)	2.8 (1.1)	2.3 (1.3)	2.8 (1.2)	<0.001^b^* PT, CT > NT	1.1 (0.2)	2.9 (1.2)	2.6 (1.4)	2.6 (1.1)	<0.001^b^* PT, IT, CT > NT
	Median (IQR)	1.0 (0.0)	3.0 (2.0)	2.0 (2.0)	3.0 (2.0)		1.0 (0.0)	3.0 (2.0)	2.0 (3.0)	3.0 (1.0)	
OBC 6. Hold, tighten, or tense muscles without clenching or bringing teeth together											
	Mean ± SD	1.6 (0.8)	2.1 (0.9)	2.0 (1.1)	2.3 (1.0)	0.124	1.1 (0.4)	2.3 (1.0)	1.6 (0.8)	1.8 (1.0)	<0.001^b^* PT, IT, CT > NT
	Median (IQR)	1.5 (1.0)	2.0 (2.0)	2.0 (2.0)	2.0 (2.0)		1.0 (0.0)	2.0 (2.0)	1.0 (1.0)	1.5 (2.0)	
OBC 7. Hold or jut jaw forward or to the side											
	Mean (SD)	1.8 (0.8)	1.8 (0.9)	1.6 (0.7)	2.3 (1.1)	0.121	1.3 (0.9)	2.0 (0.9)	1.6 (0.9)	1.9 (1.0)	<0.001^b^* PT, CT > NT
	Median (IQR)	2.0 (1.0)	2.0 (1.0)	1.5 (1.0)	2.0 (2.0)		1.0 (0.0)	2.0 (2.0)	1.0 (1.0)	2.0 (1.0)	
OBC 11. Hold jaw in rigid or tense position, such as to brace or protect the jaw											
	Mean ± SD	1.6 (0.7)	2.1 (1.0)	2.0 (0.9)	2.3 (0.9)	0.054	1.2 (0.4)	2.2 (1.0)	2.0 (1.1)	2.0 (0.9)	<0.001^b^* PT, IT, CT > NT
	Median (IQR)	1.0 (1.0)	2.0 (2.0)	2.0 (2.0)	2.0 (1.0)		1.0 (0.0)	2.0 (2.0)	2.0 (2.0)	2.0 (2.0)	
OBC-Ch 6											
	Mean (SD)	5.0 (2.8)	7.0 (3.7)	5.2 (3.9)	7.5 (3.7)	0.041*	1.4 (2.6)	7.1 (4.0)	4.9 (4.0)	5.8 (4.0)	<0.001^b^* PT, IT, CT > NT
	Median (IQR)	6.0 (4.0)	7.0 (6.0)	5.5 (6.0)	7.0 (6.0)		0.0 (2.0)	7.0 (5.0)	4.5 (6.0)	5.5 (4.0)	
OBC-Ch 8											
	Mean (SD)	5.8 (3.2)	11.3 (5.1)	9.1 (5.4)	12.4 (4.6)	<0.001^b^* PT, CT > NT	1.6 (2.8)	11.6 (4.5)	9.2 (5.7)	9.5 (5.4)	<0.001^b^* PT, IT, CT > NT
	Median (IQR)	7.0 (5.0)	12.0 (8.0)	9.0 (7.0)	13.0 (8.0)		0.0 (3.0)	12.0 (5.0)	8.5 (8.0)	9.0 (7.0)	

^b^Kruskal-Wallis-test; *Significantly different among the groups. Abbreviations: NT, no TMD; PT, pain-related TMD; IT, intra-articular TMD; CT, combined TMD; SD, standard deviation; IQR, interquartile range. Adjusted p value using Bonferroni correction.

**Table 4.** Oral Behavior Checklist (OBC) scores disaggregated by depression status.

Parameter		Depression (n = 105)					No depression (n = 175)				
		NT (n = 16)	PT (n = 33)	IT (n = 22)	CT (n = 34)	*p*-value *Post-hoc*	NT (n = 54)	PT (n = 37)	IT (n = 48)	CT (n = 36)	*p*-value *Post-hoc*
OBC 1. Clench or grind teeth when asleep, based on any information you may have											
	Mean (SD)	1.2 (0.4)	2.6 (1.5)	2.4 (1.3)	2.5 (1.5)	0.004* PT, IT, CT > NT	1.1 (0.3)	2.2 (1.2)	2.3 (1.5)	2.4 (1.6)	<0.001^b^* PT, IT, CT > NT
	Median (IQR)	1.0 (0.0)	2.0 (3.0)	2.0 (2.0)	2.0 (3.0)		1.0 (0.0)	2.0 (2.0)	2.0 (2.0)	1.0 (3.0)	
OBC 2. Sleep in a position that puts pressure on the jaw (for example, on stomach, on the side)											
	Mean (SD)	1.3 (0.4)	4.2 (1.1)	3.6 (1.7)	4.2 (1.2)	<0.001^b^* PT, IT, CT > NT	1.2 (0.5)	3.9 (1.2)	4.0 (1.5)	3.6 (1.5)	<0.001^b^* PT, IT, CT > NT
	Median (IQR)	1.0 (1.0)	5.0 (1.0)	4.5 (3.0)	5.0 (1.0)		1.0 (0.0)	4.0 (2.0)	5.0 (2.0)	4.0 (3.0)	
Sleep-related OBC total											
	Mean (SD)	0.4 (0.7)	4.8 (2.1)	4.0 (2.4)	4.6 (2.3)	<0.001^b^* PT, IT, CT > NT	0.3 (0.7)	4.2 (1.8)	4.3 (2.6)	4.0 (2.4)	<0.001^b^* PT, IT, CT > NT
	Median (IQR)	0.0 (1.0)	5.0 (2.0)	4.0 (4.3)	4.0 (3.0)		0.0 (0.0)	4.0 (3.0)	4.0 (3.0)	4.0 (4.0)	
OBC 3. Grind teeth together during waking hours											
	Mean (SD)	1.7 (0.9)	1.3 (0.7)	1.1 (0.3)	1.2 (0.5)	0.013* IT, CT > NT	1.2 (0.7)	1.4 (0.7)	1.3 (0.9)	1.3 (0.6)	0.261
	Median (IQR)	1.5 (1.0)	1.0 (0.0)	1.0 (0.0)	1.0 (0.0)		1.0 (0.0)	1.0 (1.0)	1.0 (0.0)	1.0 (0.0)	
OBC 4. Clench teeth together during waking hours											
	Mean (SD)	2.9 (1.7)	2.7 (1.1)	2.2 (1.0)	2.4 (1.3)	0.267	1.7 (1.3)	2.4 (1.1)	1.8 (1.0)	2.3 (1.2)	<0.001^b^* PT, CT > NT
	Median (IQR)	2.5 (4.0)	3.0 (2.0)	2.0 (2.0)	2.0 (2.0)		1.0 (1.0)	2.0 (2.0)	1.5 (1.0)	2.0 (2.0)	
OBC 5. Press, touch, or hold teeth together other than while eating (that is, contact between upper and lower teeth)											
	Mean (SD)	1.2 (0.4)	2.9 (1.2)	2.1 (1.1)	2.7 (1.0)	<0.001^b^* PT, CT > NT	1.1 (0.2)	2.8 (1.1)	2.7 (1.5)	2.8 (1.3)	<0.001^b^* PT, IT, CT > NT
	Median (IQR)	1.0 (0.0)	3.0 (2.0)	2.0 (2.0)	3.0 (1.0)		1.0 (0.0)	3.0 (2.0)	2.5 (3.0)	3.0 (2.0)	
OBC 6. Hold, tighten, or tense muscles without clenching or bringing teeth together											
	Mean ± SD	1.4 (0.5)	2.2 (1.0)	1.8 (0.8)	2.2 (1.0)	0.007* PT, CT > NT	1.2 (0.5)	2.3 (1.0)	1.7 (0.9)	1.8 (1.0)	<0.001^b^* PT, IT, CT > NT; PT > IT
	Median (IQR)	1.0 (1.0)	2.0 (2.0)	2.0 (1.0)	2.0 (2.0)		1.0 (0.0)	2.0 (2.0)	1.0 (1.0)	1.5 (2.0)	
OBC 7. Hold or jut jaw forward or to the side											
	Mean (SD)	1.6 (0.7)	1.9 (0.9)	1.8 (1.0)	2.3 (1.1)	0.127	1.3 (0.9)	1.9 (0.9)	1.5 (0.7)	1.9 (1.0)	<0.001^b^* PT, CT > NT
	Median (IQR)	1.5 (1.0)	2.0 (1.0)	2.0 (1.0)	2.0 (2.0)		1.0 (0.0)	2.0 (2.0)	1.0 (1.0)	2.0 (2.0)	
OBC 11. Hold jaw in rigid or tense position, such as to brace or protect the jaw											
	Mean ± SD	1.4 (0.6)	2.4 (1.1)	1.9 (0.8)	2.4 (1.0)	0.001* PT, CT > NT	1.2 (0.5)	2.0 (1.0)	2.1 (1.2)	1.9 (0.9)	<0.001^b^* PT, IT, CT > NT
	Median (IQR)	1.0 (1.0)	3.0 (2.0)	2.0 (1.0)	2.0 (1.0)		1.0 (0.0)	2.0 (2.0)	2.0 (2.0)	2.0 (2.0)	
OBC-Ch 6											
	Mean (SD)	4.2 (3.1)	7.3 (3.9)	4.8 (3.0)	7.1 (3.7)	0.004* PT > NT	1.6 (2.8)	6.8 (3.9)	5.1 (4.3)	6.0 (4.1)	<0.001^b^* PT, IT, CT > NT
	Median (IQR)	5.0 (6.0)	8.0 (6.0)	5.5 (4.0)	7.0 (6.0)		0.0 (3.0)	7.0 (5.0)	5.0 (7.0)	6.0 (7.0)	
OBC-Ch 8											
	Mean (SD)	4.6 (3.4)	12.1 (4.9)	8.8 (4.2)	11.7 (4.9)	<0.001^b^* PT, CT > NT	1.9 (3.1)	10.9 (4.5)	9.4 (6.2)	10 (5.5)	<0.001^b^* PT, IT, CT > NT
	Median (IQR)	5.0 (7.0)	14.0 (7.0)	9.0 (6.0)	12.0 (6.0)		0.0 (3.0)	11.0 (5.0)	8.5 (9.0)	9.0 (6.0)	

^b^Kruskal-Wallis-test; *Significantly different among the groups. Abbreviations: NT, no TMD; PT, pain-related TMD; IT, intra-articular TMD; CT, combined TMD; SD, standard deviation; IQR, interquartile range. Adjusted p value using Bonferroni correction.

As shown in Table 3, the OBC-Ch 6 and OBC-Ch 8 scores of non-anxious subjects (n = 181) were the highest in the PT group (median: 7, 12) and the lowest in the NT group (median: 0, 0). The PT, IT and CT groups had scores that were significantly higher than the NT group, as assessed by *post hoc* pair-wise comparisons (*p* < 0.001). However, among anxious subjects (n = 99), the highest OBC-Ch 6 and OBC-Ch 8 scores were those of the CT group (median: 7, 13). Moreover, the PT group and CT group had significantly higher scores than the NT group, as assessed by *post hoc* pair-wise comparisons of the anxious subjects in each group (*p* < 0.001).

Table 4 shows the OBC scores of participants with and without depression in each group. Significant differences were observed in OBC-Ch 6 (PT, IT, CT > NT) and OBC-Ch 8 (PT, IT, CT > NT) scores across groups in non-depressed participants (n = 175). In depressed participants (n = 105), significant differences were also observed in OBC-Ch 6 (PT > NT) and OBC-Ch 8 (PT, CT > NT).

## 4. Discussion

In the present study, we aimed to determine the distribution of various oral behaviors and the prevalence of bruxism, as well as the role of psychological distress like anxiety and depression, that may affect patients with various TMD subtypes based on self-reported questionnaires.

In this study, we utilized self-reporting measures combined with physical examination to reinforce the accurate diagnosis of TMD subtypes. The 5Ts screening questionnaire is a validated tool for the identification of individual TMD status. In addition, we specifically assessed awake and asleep oral behaviors based on the OBC, which has also been validated in previous studies [7, 30].

Several studies have indicated that various oral behaviors are possible contributors to TMDs [8, 31]. The prolonged loading caused by oral activities have been associated with dysfunction of the masticatory muscles and a high stress distribution in the disc, leading to TMDs [32]. Previous studies have found a variety of associations, or lack thereof, between awake and sleep-related oral behaviors and specific TMD subtypes [11]. In the present study, we analyzed participants’ responses to OBC items focused on oral behaviors, including both awake and sleep-related oral activities, to comprehensively investigate the distribution of oral behaviors in different TMD subgroups. The results showed significant differences in OBC-Ch 8, OBC-Ch 6 and asleep oral behavior scores among the three subtypes of TMDs studied here and the control group without TMDs. Intergroup comparisons revealed that participants with TMDs had significantly higher scores than controls in the NT group, similar to previous studies [30, 33]. Overall, scores tended to be higher in the current study, which may be due to the sample with only female participants enrolled.

The OBC scores were higher in the PT group and CT group among the different TMD subgroups evaluated in this study, which is in line with the findings of previous studies [33, 34]. The scores on assessments of awake and sleep-related oral behaviors in the IT group were lower than those of the PT group and CT group, again in agreement with previous studies [14, 35]. Sun *et al*. [28] found that the PT patient have higher frequency oral behaviors than those without PT subjects in general participants with the same OBC scale. Indeed, participants with dysfunctional TMDs showed a lower frequency of oral behaviors compared to those with painful TMDs. Frequent oral behaviors are a known risk factor for painful TMDs [36]. Chow *et al*. [6] reported that participants with facial pain have more frequent oral behaviors than those without facial pain. Barbosa *et al*. [8] studied young university students and found that those with higher levels of overuse of oral behaviors had a stronger association with painful TMDs compared to students with fewer and less-frequent behaviors, and the level of overuse of oral behaviors exhibited a dose-response relationship with the severity of TMD-associated facial pain. Moreover, Keela *et al*. [9] investigated the OBC scores associated with various types of pain in TMDs. They reported that high OBC scores were associated with chronic painful TMDs, and suggested that managing oral behaviors during TMD treatment is warranted. However, Lövgren *et al*. [10] evaluated the association between oral behaviors and functional jaw disturbances, and they reported no significant association between the frequency of oral behaviors and a positive screening for functional jaw disturbances. The differences in these results might be due to differing validity of OBC items, which may be influenced by social or cultural factors that vary in different regions [21].

A considerable amount of research has established that age is one of the factors contributing to differences in the distribution of TMD subgroups. In this study, adult subjects were included with a mean age of 31.29 ± 10.36, which was different from the research by Adrian *et al*. [30]. The participants in their study were younger university students with a mean age of 22.7 ± 1.1, which could have contributed to the difference in results. Moreover, Michelotti *et al*. [33] found that female patients have a significant risk of myofascial pain, while the risk of developing disc displacement decreases with their age.

Bruxism, long considered a disorder or pathology, is now thought to be a motor activity that may be a potential risk factor for some diseases and may even have possible physiological or protective relevance [37]. The association of TMDs with self-reported frequent AB and SB with is a controversial topic. In this study, TMD patients were significantly more likely than participants in the NT group to report SB, whereas we found no significant differences in AB across groups. Su *et al*. [38] found that self-reported AB and SB are both associated with lower oral health-related quality of life in TMD patients. Similar to previous reported findings, in our study the prevalence of AB was about 30% in our sample of female adults [39], and the AB distribution was not statistically different between TMD subtypes or in NT controls. The differing degrees of SB prevalence in our results were in line with previous studies which indicated that SB is associated with TMDs [40]. Nevertheless, several studies have suggested that it is not possible to draw definitive conclusions about whether there may be a cause-effect relationship between SB and TMDs. The apparent differences in bruxism rates might indicate that self-reporting of sleep bruxism is unreliable, and varying findings in the literature could also be due to the heterogeneity of study designs [41]. Thus, it has been recommended to assess oral conditions related to the spectrum of bruxism activities using measures other than self-reporting, such as electromyographic (EMG) or polysomnographic (PSG) instrumental measures [42].

Psychological disorders such as anxiety and depression have been correlated with the development of TMDs and inherent comorbidity [43]. Zlendić *et al*. [11] observed differences in psychological variables (anxiety and depression) between their control group and patients with TMDs, especially in the higher pain intensity TMD group. Nevertheless, Yao *et al*. [44]. investigated the mental health of patients with reversible anterior disc displacement, and they found that the measures of anxiety and depression were not significantly different in these patients compared to the control group. Adding nuance to this picture, oral behaviors and psychological distress have been differentially associated with particular TMD symptoms. Thus, it might be necessary to assess the distribution of psychological stress (anxiety and depression) and oral behaviors among different TMD subgroups.

Since psychosocial factors are known to be related to both oral behaviors and pain intensity, they deserve to be the focus of further research in the future. More precise and detailed research projects will clarify the potentially bidirectional relationships between oral behaviors, pain, and psychosocial factors. According to our findings in the present study, anxiety and depression were significantly more prevalent in the PT group and CT groups, both TMD subtypes that involve a pain component, than in the NT group or the non-painful IT group. Our results indicated that oral behavior scores for both awake and asleep activities and global scores of OBC-Ch 8 were higher in individuals suffering from anxiety and depression, consistent with previous studies [6, 45]. Furthermore, individuals diagnosed with anxiety or depression in the NT group had higher oral behavior scores than others in the NT group. Therefore, assessments of patients’ individual psychological status to identify anxiety and depression is recommended before treatment of TMDs.

Most TMDs have a good prognosis. However, in some specific TMD patients with disease that involves a serious Axis-I or Axis-II component, clinical experts suggest multi-disciplinary treatment by dental and medical specialists, such as orofacial pain specialists, TMJ surgeons, psychologists and psychiatrists [46]. This approach may particularly be particularly important for patients for whom a painful TMD is associated with anxiety and/or depression.

The TMD diagnoses of patients in our study were confirmed through self-reported questionnaires and physical examination, ensuring highly reliable classification. However, this study still has certain limitations. First, this was a case-control study of patients recruited from one clinical center, with only female participants. We did not interrogate or speculate on cause-effect relationships in this study. Moreover, the female sample size in the study was limited and the results may not represent the general population. Further study should include a larger sample size representing a broader spectrum of genders and ages. The use of standardized tools is encouraged to assess bruxism, and instrumental measures like PSG data during a sleep study might be utilized for more accurate assessment of various oral behaviors as well as definition of sleep bruxism. In addition, the 5Ts scale, although concise and easy to implement, cannot reflect the severity of TMDs. A more comprehensive clinical examination including imaging data and more detailed questionnaires may be required in future studies to accurately assess the relationship between oral behaviors, psychological stress and TMDs.

## 5. Conclusions

In this study, we found that TMD patients present significantly higher frequency of oral behaviors than healthy subjects. This was especially true of patients experiencing painful TMDs and psychological distress. Clinicians should pay attention to special oral behaviors in patients with TMDs. Interventions for oral behaviors in patients with pain, especially pain combined with psychological distress, may have additional benefits. Further prospective researches are necessary to clarify causal mechanisms and effects of oral behaviors on TMDs outcomes.

## References

[1] Xie C, Lin M, Yang H, Ren A. Prevalence of temporomandibular disorders and its clinical signs in Chinese students, 1979–2017: a systematic review and meta-analysis. Oral Diseases. 2019; 25: 1697–1706. 10.1111/odi.13016 30548954

[2] Kapos FP, Exposto FG, Oyarzo JF, Durham J. Temporomandibular disorders: a review of current concepts in aetiology, diagnosis and management. Oral Surgery. 2020; 13: 321–334. 10.1111/ors.12473 PMC8631581 34853604

[3] Scrivani SJ, Keith DA, Kaban LB. Temporomandibular disorders. The New England Journal of Medicine. 2008; 359: 2693–2705. 10.1056/NEJMra0802472 19092154

[4] Yi Y, Zhou X, Xiong X, Wang J. Neuroimmune interactions in painful TMD: Mechanisms and treatment implications. Journal of Leukocyte Biology. 2021; 110: 553–563. 10.1002/JLB.3MR0621-731RR 34322892

[5] Warzocha J, Gadomska-Krasny J, Mrowiec J. Etiologic factors of temporomandibular disorders: a systematic review of literature containing diagnostic criteria for temporomandibular disorders (DC/TMD) and research diagnostic criteria for temporomandibular disorders (RDC/TMD) from 2018 to 2022. Healthcare. 2024; 12: 575. 10.3390/healthcare12050575 PMC10931313 38470686

[6] Chow JC, Cioffi I. Effects of trait anxiety, somatosensory amplification, and facial pain on self-reported oral behaviors. Clinical Oral Investigations. 2019; 23: 1653–1661. 10.1007/s00784-018-2600-1 30151704

[7] Tang Y, Fan S, Yao Y, Xu LL, Cai B. Oral behavior characteristics of 540 patients suffering from temporomandibular disorders. Shanghai Journal of Stomatology. 2021; 30: 531–534. (In Chinese) 34888608

[8] Barbosa C, Manso MC, Reis T, Soares T, Gavinha S, Ohrbach R. Are oral overuse behaviours associated with painful temporomandibular disorders? A cross-sectional study in Portuguese university students. Journal of Oral Rehabilitation. 2021; 48: 1099–1108. 10.1111/joor.13226 34273189

[9] Keela W, Itthikul T, Mitrirattanakul S, Pongrojpaw S. Awake and sleep oral behaviours in patients with painful temporomandibular disorders. International Dental Journal. 2024; 74: 138–145. 10.1016/j.identj.2023.07.013 PMC10829361 37586995

[10] Lövgren A, Ilgunas A, Häggman-Henrikson B, Elias B, Roudini OA, Visscher CM, *et al*. Associations between screening for functional jaw disturbances and patient reported outcomes on jaw limitations and oral behaviors. Journal of Evidence-Based Dental Practice. 2023; 23: 101888. 10.1016/j.jebdp.2023.101888 37689443

[11] Zlendić M, Vrbanović E, Tomljanović M, Gall Trošelj K, Đerfi KV, Alajbeg IZ. Association of oral behaviours and psychological factors with selected genotypes in pain-related TMD. Oral Diseases. 2024; 30: 1702–1715. 10.1111/odi.14583 37036392

[12] Markiewicz MR, Ohrbach R, McCall WD Jr. Oral behaviors checklist: reliability of performance in targeted waking-state behaviors. Journal of Orofacial Pain. 2006; 20: 306–316. 17190029

[13] Slade GD, Fillingim RB, Sanders AE, Bair E, Greenspan JD, Ohrbach R, *et al*. Summary of findings from the OPPERA prospective cohort study of incidence of first-onset temporomandibular disorder: implications and future directions. The Journal of Pain. 2013; 14: T116–T124. 10.1016/j.jpain.2013.09.010 PMC3857103 24275219

[14] Donnarumma V, Ohrbach R, Simeon V, Lobbezoo F, Piscicelli N, Michelotti A. Association between waking-state oral behaviours, according to the oral behaviors checklist, and TMD subgroups. Journal of Oral Rehabilitation. 2021; 48: 996–1003. 10.1111/joor.13221 PMC8457156 34192368

[15] Suvinen TI, Reade PC, Kemppainen P, Könönen M, Dworkin SF. Review of aetiological concepts of temporomandibular pain disorders: towards a biopsychosocial model for integration of physical disorder factors with psychological and psychosocial illness impact factors. The Journal of Pain. 2005; 9: 613–633. 10.1016/j.ejpain.2005.01.012 15978854

[16] Salinas Fredricson A, Krüger Weiner C, Adami J, Rosén A, Lund B, Hedenberg-Magnusson B, *et al*. The role of mental health and behavioral disorders in the development of temporomandibular disorder: a SWEREG-TMD nationwide case-control study. Journal of Pain Research. 2022; 15: 2641–2655. 10.2147/JPR.S381333 PMC9464023 36097536

[17] Wu Y, Xiong X, Fang X, Sun W, Yi Y, Liu J, *et al*. Psychological status of TMD patients, orthodontic patients and the general population during the COVID-19 pandemic. Psychology, Health & Medicine. 2021; 26: 62–74. 10.1080/13548506.2020.1858489 33347345

[18] Dan R, Li J, Xie T, Luo M, Lau RS, Hu S, *et al*. Impact of different types of temporomandibular disorders on jaw functional limitation and psychological distress in orthodontic patients. Journal of Oral Rehabilitation. 2023; 50: 644–654. 10.1111/joor.13463 37036764

[19] Zheng Y, Zhou X, Huang Y, Lu J, Cheng Q, Fan P, *et al*. Low income is associated with impaired jaw function via anxiety and depression in patients with temporomandibular disorders. Journal of Oral Rehabilitation. 2023; 50: 1373–1381. 10.1111/joor.13579 37641469

[20] Ye C, Xiong X, Zhang Y, Pu D, Zhang J, Du S, *et al*. Psychological profiles and their relevance with temporomandibular disorder symptoms in preorthodontic patients. Pain Research & Management. 2022; 2022: 1039393. 10.1155/2022/1039393 PMC9553652 36247102

[21] Donnarumma V, Cioffi I, Michelotti A, Cimino R, Vollaro S, Amato M. Analysis of the reliability of the Italian version of the oral behaviours checklist and the relationship between oral behaviours and trait anxiety in healthy individuals. Journal of Oral Rehabilitation. 2018; 45: 317–322. 10.1111/joor.12614 29420851

[22] Lobbezoo F, Ahlberg J, Raphael KG, Wetselaar P, Glaros AG, Kato T, *et al*. International consensus on the assessment of bruxism: report of a work in progress. Journal of Oral Rehabilitation. 2018; 45: 837–844. 10.1111/joor.12663 PMC6287494 29926505

[23] Manfredini D, Lobbezoo F. Sleep bruxism and temporomandibular disorders: a scoping review of the literature. Journal of Dentistry. 2021; 111: 103711. 10.1016/j.jdent.2021.103711 34090993

[24] Ohlmann B, Waldecker M, Leckel M, Bömicke W, Behnisch R, Rammelsberg P, *et al*. Correlations between sleep bruxism and temporomandibular disorders. Journal of Clinical Medicine. 2020; 9: 611. 10.3390/jcm9020611 PMC7074179 32102466

[25] Bartolucci ML, Incerti Parenti S, Bortolotti F, Della Godenza V, Vandi S, Pizza F, *et al*. Sleep bruxism and orofacial pain in patients with sleep disorders: a controlled cohort study. Journal of Clinical Medicine. 2023; 12: 2997. 10.3390/jcm12082997 PMC10142632 37109339

[26] Yap AU, Dworkin SF, Chua EK, List T, Tan KB, Tan HH. Prevalence of temporomandibular disorder subtypes, psychologic distress, and psychosocial dysfunction in Asian patients. Journal of Orofacial Pain. 2003; 17: 21–28. 12756927

[27] Yap AU, Zhang MJ, Zhang XH, Cao Y, Fu KY. Viability of the quintessential 5 temporomandibular disorder symptoms as a TMD screener. Oral Surgery, Oral Medicine, Oral Pathology and Oral Radiology. 2022; 133: 643–649. 10.1016/j.oooo.2021.11.009 35153185

[28] Sun R, Zhang S, Si J, Zhang L, Yang H, Ye Z, *et al*. Association between oral behaviors and painful temporomandibular disorders: a cross-sectional study in the general population. Journal of Pain Research. 2024; 17: 431–439. 10.2147/JPR.S449377 PMC10848921 38328021

[29] Knibbe W, Lobbezoo F, Voorendonk EM, Visscher CM, de Jongh A. Prevalence of painful temporomandibular disorders, awake bruxism and sleep bruxism among patients with severe post-traumatic stress disorder. Journal of Oral Rehabilitation. 2022; 49: 1031–1040. 10.1111/joor.13367 PMC9825945 36056716

[30] Yap AU, Marpaung C. Personality, psychosocial and oral behavioural risk factors for temporomandibular disorder symptoms in Asian young adults. Journal of Oral Rehabilitation. 2023; 50: 931–939. 10.1111/joor.13527 37256928

[31] Medin Ceylan C, Cigdem Karacay B. The relationship between the oral behavioral checklist and the jaw functional limitation scale in temporomandibular joint pain. BMC Oral Health. 2023; 12: 112–117.

[32] Barrientos E, Pelayo F, Tanaka E, Lamela-Rey MJ, Fernández-Canteli A, de Vicente JC. Effects of loading direction in prolonged clenching on stress distribution in the temporomandibular joint. Journal of the Mechanical Behavior of Biomedical Materials. 2020; 112: 104029. 10.1016/j.jmbbm.2020.104029 32827997

[33] Michelotti A, Cioffi I, Festa P, Scala G, Farella M. Oral parafunctions as risk factors for diagnostic TMD subgroups. Journal of Oral Rehabilitation. 2010; 37: 157–162. 10.1111/j.1365-2842.2009.02033.x 20002533

[34] Glaros AG, Williams K, Lausten L. The role of parafunctions, emotions and stress in predicting facial pain. The Journal of the American Dental Association. 2005; 136: 451–458. 10.14219/jada.archive.2005.0200 15884314

[35] Glaros AG, Williams K, Lausten L, Friesen LR. Tooth contact in patients with temporomandibular disorders. CRANIO®. 2005; 23: 188–193. 10.1179/crn.2005.027 16128353

[36] Ohrbach R, Sharma S. Temporomandibular disorders: definition and etiology. Seminars in Orthodontics. 2023: 30: 237–242.

[37] Manfredini D, Ahlberg J, Aarab G, Bender S, Bracci A, Cistulli PA, *et al*. Standardised tool for the assessment of bruxism. Journal of Oral Rehabilitation. 2024; 51: 29–58. 10.1111/joor.13411 36597658

[38] Su N, Liu Y, Yang X, Shen J, Wang H. Association of malocclusion, self-reported bruxism and chewing-side preference with oral health-related quality of life in patients with temporomandibular joint osteoarthritis. International Dental Journal. 2018; 68: 97–104. 10.1111/idj.12344 PMC9378935 29094335

[39] Melo G, Duarte J, Pauletto P, Porporatti AL, Stuginski-Barbosa J, Winocur E, *et al*. Bruxism: an umbrella review of systematic reviews. Journal of Oral Rehabilitation. 2019; 46: 666–690. 10.1111/joor.12801 30993738

[40] Ekman A, Rousu J, Näpänkangas R, Kuoppala R, Raustia A, Sipilä K. Association of self-reported bruxism with temporomandibular disorders—Northern Finland Birth Cohort (NFBC) 1966 study. CRANIO®. 2023; 41: 212–217. 10.1080/08869634.2020.1853306 33267744

[41] Raphael KG, Sirois DA, Janal MN, Wigren PE, Dubrovsky B, Nemelivsky LV,* et al*. Sleep bruxism and myofascial temporomandibular disorders: a laboratory-based polysomnographic investigation. The Journal of the American Dental Association. 2012; 143: 1223–1231. 10.14219/jada.archive.2012.0068 PMC4480769 23115152

[42] Manfredini D, Ahlberg J, Aarab G, Bracci A, Durham J, Emodi-Perlman A, *et al*. The development of the Standardised Tool for the Assessment of Bruxism (STAB): an international road map. Journal of Oral Rehabilitation. 2024; 51: 15–28. 10.1111/joor.13380 36261916

[43] Reis PHF, Laxe LAC, Lacerda-Santos R, Münchow EA. Distribution of anxiety and depression among different subtypes of temporomandibular disorder: a systematic review and meta-analysis. Journal of Oral Rehabilitation. 2022; 49: 754–767. 10.1111/joor.13331 35398904

[44] Yao Y, Liu SS, Jin L, Zeng H, Jiang X, Fang ZY, *et al*. Mental health and jaw function of patients with anterior disc displacement with reduction. Journal of Oral Rehabilitation. 2024; 51: 677–683. 10.1111/joor.13637 38087998

[45] Xu L, Cai B, Fan S, Lu S, Dai K. Association of oral behaviors with anxiety, depression, and jaw function in patients with temporomandibular disorders in china: a cross-sectional study. Medical Science Monitor. 2021; 27: e929985. 10.12659/MSM.929985 PMC8139132 33999914

[46] Patel K, Eley KA, Cascarini L, Watt-Smith S, Larkin M, Lloyd T, *et al*. Temporomandibular disorders-review of evidence-based management and a proposed multidisciplinary care pathway. Oral Surgery, Oral Medicine, Oral Pathology and Oral Radiology. 2023; 136: 54–69. 10.1016/j.oooo.2023.02.001 36990844

